# Social communication in fragile X syndrome: pilot examination of the Brief Observation of Social Communication Change (BOSCC)

**DOI:** 10.1186/s11689-021-09411-z

**Published:** 2022-01-08

**Authors:** Rebecca Shaffer, Angela John Thurman, Lucienne Ronco, Diego Cadavid, Shane Raines, So Hyun Kim

**Affiliations:** 1 Department of Pediatrics, Cincinnati Children’s Hospital Medical Center, University of Cincinnati School of Medicine, 3333 Burnet Avenue, MLC 4002, Cincinnati, OH 45229 USA; 2 Department of Psychiatry and Behavioral Sciences, University of California Davis Health, MIND Institute, University of California Davis Health, Sacramento, CA USA; 3 Fulcrum Therapeutics, Cambridge, MA USA; 4 Deep Genomics Therapeutics, Toronto, ON Canada; 5 University of Massachusetts Medical School, Worcester, MA USA; 6 2b Analytics, Wallingford, PA USA; 7 Department of Psychiatry, Weill Cornell Medicine, New York, NY USA

**Keywords:** Fragile X syndrome, BOSCC, Social communication, Repetitive behaviors, Outcome measure

## Abstract

**Background:**

Social communication is a key area of difficulty in fragile X syndrome (FXS) and there are not yet adequate outcome measurement tools. Appropriate outcome measures for FXS have been identified as a key area of research interest in order to evaluate future therapeutic trials. The Brief Observation of Social Communication Change-Minimally Verbal (BOSCC-MV), an outcome measure with strong psychometrics developed for autism spectrum disorder, has promise as an outcome measure to assess social communication change with FXS participants.

**Methods:**

We examined the BOSCC-MV via central coders in this multi-site-trial to assess its appropriateness for FXS. Eighteen minimally verbal males ages 3–12 years were enrolled and assessed on two consecutive days and 7 participants completed a third visit 6 months later. We examined test-retest reliability, inter-rater reliability, and both convergent and divergent validity with standard clinical measures including the Autism Diagnostic and Observation Schedule-2, Vineland 3, Social Responsiveness Scale, and the Aberrant Behavior Checklist.

**Results:**

The BOSCC-MV in FXS demonstrated strong inter-rater and test-retest reliability, comparable to previous trials in idiopathic ASD. Strong convergent validity was found with Autism Diagnostic Observation Schedule-2 and Vineland-3. Divergent validity was demonstrated between BOSCC-MV and unrelated measures.

**Conclusions:**

The BOSCC-MV shows promise as a FXS social communication outcome measure, warranting further large-scale evaluation.

## Background

Fragile X syndrome (FXS) is the most common inherited, genetic cause of intellectual disability in the world [42]. This serious neurodevelopmental disorder is caused by missing or insufficient production of fragile X mental retardation protein (FMRP) in neurons in the brain resulting from expanded CGG repeats on intron 1 of the FMR1 gene on the X chromosome [42]. Males tend to be more significantly impaired than females, given the presence of only one X chromosome [1, 41]. The clinical manifestations of FXS are diverse and vary from mild to severe intellectual disability with variable behavioral impairments. Nonetheless, key areas of impairment commonly associated with the FXS phenotype include social communication (SC) difficulties, expressive language delays, restricted and repetitive behaviors (RRBs), and increased rates of social anxiety, attention deficit with hyperactivity, and sensory hypersensitivity [5, 46]. Moreover, FXS is the most common single-gene cause of autism spectrum disorder (ASD), which is posited to be due to neurobiological similarities across other causes of ASD and FXS. Indeed, not only are many of the ASD susceptibility genes identified to date controlled by FMRP (e.g., SHANK and PAK), but also abnormalities in the GABAergic signaling system have been implicated in both FXS and ASD [8].

SC is defined as the use of verbal and non-verbal skills for social interaction and includes use of appropriate eye contact, facial expressions, social overtures, requesting, joint attention, and gestures. SC impairments are universally present in FXS. For example, early difficulties in joint attention and requesting, gesture use, and using gaze and emotional cues in word learning are commonly observed in children with FXS ([3, 13, 20, 35]; Thurman, McDuffie, Kover, Hagerman, Channell, et al. 2015). Furthermore, SC difficulties continue into adulthood, with pragmatic language difficulties often noted in males with FXS, including frequent use of perseverative language and tangential speech as well as difficulties with turn-taking, topic maintenance, and fluency [30, 33, 41, 44, 47, 51]. These lifelong SC difficulties can disrupt not only reciprocal social interactions, but also impede the development of structural language skills, a key area of concern among families of individuals with FXS [57]. SC difficulties impact abilities to form friendships and successfully engage in school and work settings, eventually impacting overall quality of life and, thus, is a high priority intervention target.

In addition to SC impairments, RRBs are a common area of difficulty for individuals with FXS. Examples include hand flapping, body rocking, self-injurious behaviors, and verbal perseveration [22, 36, 40]. In comparison with other genetic syndromes, individuals with FXS demonstrate more RRBs [40] and in comparison, to youth with idiopathic ASD, individuals with co-occurring FXS and ASD demonstrate equal amounts of less complex forms of RRB’s such as body and hand movements [59]. A recent study across a wide age range in FXS found that sensory related RRBs are more common in young children but other forms of RRBs tend to remain stable across ages [43]. Additionally, caregivers reported most concerns with transition difficulties and hand and finger mannerisms. Given its prevalence and uniqueness to FXS in comparison with other syndromes, RRB is a common target in FXS trials. However, current measurement tools for RRBs are limited to parent report, highlighting the need for the development of a more objective observational measurement approach.

There are no approved treatments for FXS, and very few therapeutic clinical trials in FXS have been conducted with positive results by either industry or academic investigators [4, 6, 19, 24, 60]. Positive trials have focused primarily on co-occurring behavior difficulties [23], language interventions [37, 56], and oxytocin which demonstrated improved eye contact [21]. Working groups have noted that a major obstacle impeding the success of clinical trials in FXS has been a lack of sensitive and appropriate outcome measures to test the therapeutic efficacy of FXS drug candidates and behavioral interventions [6, 24]. Despite their clinical significance, there are known limitations associated with tools considered to date as outcome measures of SC and RRBs. For example, self-report tools are largely unavailable for young children or children with limited language skills. In addition, caregiver reports are known to be affected by strong placebo effects and are often not sensitive to detect subtle changes in SC over time in this population [6, 27]. Because ameliorating these symptoms early on is crucial for both social success and the development of language skills, there is a high unmet need for validated outcome measures to assess changes in SC and RRBs in FXS.

The Brief Observation of Social Communication Change (BOSCC), a new outcome measure for SC and RRBs [16, 31], was developed with the specific goal of capturing longitudinal changes in an objective and standardized way for children with idiopathic ASD. The BOSCC has been applied to multiple videotaped interactions including play with caregivers or blind research personnel in both home and lab settings [16, 29], ADOS administrations with a blind examiner [28], and snack interactions with caregivers [14]. The flexibility of setting and administration options is ideal for application across a variety of study types. The version for minimally verbal (MV) children, BOSCC-MV, has been validated in children with idiopathic ASD, demonstrating high to excellent inter-rater and test-retest reliability and convergent validity with other measures of language and communication skills in the idiopathic ASD population [14, 16, 17, 28, 29]. The BOSCC has shown sensitivity to detect changes in a relatively short period of time (e.g., as short as 12 weeks) and to be more sensitive than the Autism Diagnostic and Observation Schedule-2 (ADOS-2) in capturing SC changes over time [28]. The BOSCC-MV coding scheme was created utilizing and expanding scoring from the ADOS-2. It involves a decision tree that guides the rater to the final code, which facilitates clear and objective determination of a final code. Therefore, the BOSCC-MV exhibits many appealing features that warrant further development and evaluation as a possible outcome measure for SC and RRBs with MV children in FXS therapeutic clinical trials. Although previous findings regarding the use of the BOSCC-MV in individuals with idiopathic ASD suggest this is a promising option for use in treatment trials, the psychometric properties of the BOSCC-MV have not yet been studied in individuals with FXS specifically [6, 24]. Indeed, although there are behavioral and neurobiological similarities between the FXS and idiopathic ASD phenotypes, key differences are noted as well. For example, compared to their male peers with idiopathic ASD, males with FXS demonstrate, on average, a milder presentation of ASD, more significant cognitive delays, more impaired language skills, and increased rates of attentional difficulties and anxiety symptomatology [15, 38, 52, 53, 55, 59].

The goal of this study was to explore the utility of the BOSCC-MV as an outcome measure of SC and RRBs in minimally verbal children with FXS in a multicenter clinical trial setting using central coders. To our knowledge, this is the first study aimed to examine feasibility, reliability, and validity of the BOSCC-MV in this population. In addition, the study sought to evaluate any need to optimize the current BOSCC-MV administration or coding algorithm for use in subjects with FXS. This pilot validation study focused on males ages 3–12 with FXS with minimal verbal language, as they represent the most impaired FXS group with the highest unmet need and the most likely to benefit from treatments. It was completed as a collaboration between academic investigators and clinical researchers at Fulcrum Therapeutics to further develop appropriate outcome measures for FXS.

## Methods

### Study design

This was a multicenter initial validation study of the BOSCC-MV as an outcome measure of SC and RRBs in minimally verbal children with FXS (i.e., no words to short, inflexible phrases only). IRB approval was obtained at all sites. Following pre-screening to determine study eligibility, the subjects were assessed in the clinic during a minimum of two study visits that were 1 day apart to measure test/retest reliability, convergent and divergent validity versus other outcome measures, and multi-site feasibility. Although a single day in between visits is shorter than the typical week utilized in similar studies, a day was chosen to decrease burden for families as many were traveling from a distance to the appointment and were unable to stay for an entire week versus two consecutive days. There was an optional third visit to measure sensitivity to change over approximately 6 months. The third visit was made optional, given many families were traveling for the study and unable to return for the follow up visit. Each time point involved BOSCC-MV administration and other parent reported, or clinician administered measures. Each BOSCC-MV administration was video recorded for coding of the play session after the visit. Informed consent was obtained from caregivers in the first visit. Assent was not obtained in this study given the participants’ minimal verbal ability.

Male subjects 3 to 12 years of age with a medically documented genetic report of FXS and English as their primary language were included in this study. Children were minimally verbal, with language clearly below chronological age expectations based on the appropriateness of a ADOS Module 1 administration (i.e., mainly no words to single words only, without flexible 2- to 3-word phrases). It should be noted our final sample only included 3–8-year-old children. All children screened between the ages of 9–12 were excluded due to more advanced language. Participants were recruited from the local community and families receiving clinical services at two participating sites in the United States. For those receiving other services who qualified for the project, a research coordinator contacted the family to share information about the study and assess interest in participating. Children were excluded from participation if sensory impairments (i.e., deafness or blindness) would interfere with the valid administration of study measures. Non-English-speaking children were excluded since we did not have available examiners or coders who could speak languages other than English.

Overall, 18 subjects were enrolled in the study with all subjects (100%) completing the study. All children enrolled in the study were able to complete the BOSCC-MV at the first visit. The median overall age of subjects was 4.0 years (range 3 to 8 years; Table 1). All enrolled subjects (100.0%) were male. The mean IQ for the group was 57.6 ± 15.8 (range = 47–103). The enrolled population was predominantly Caucasian or White (66.7%) and not Hispanic or Latino (94.4%). All subjects (100%) in the enrolled analysis set were confirmed with a genetic diagnosis of FXS. Three subjects (16.7%) were confirmed as positive for mosaicism while 7 subjects (38.9%) were test-negative and 8 subjects (44.4%) had unknown results. Demographics for the smaller group who returned for the third visit are also reported in Table 1.

**Table 1:** Demographics characteristics and clinical measures

	Visit 1 Overall (*N* = 18)	Visit 3 Overall (*N* = 7)
**Age at consent (years)**		
Mean (SD)	4.5 (1.54)	5.3 (1.60)
**Sex**		
Male, *n* (%)	18 (100.0%)	7 (100%)
**Race**		
African American or Black, *n* (%)	1 (5.6%)	0
Caucasian or White, *n* (%)	12 (66.7%)	7 (100%)
More than one race, *n* (%)	5 (27.8%)	0
**Ethnicity**		
Hispanic/Latino, *n* (%)	1 (5.6%)	0
Not Hispanic or Latino, *n* (%)	17 (94.4%)	7 (100%)
**Stanford Binet abbreviated IQ**	57.6 (15.8, range = 47–103)	63.6 (20.35, range = 47–103)
Verbal scaled score	2.6 (2.23, range = 1–8)	3.1 (2.12, range = 1–6)
Nonverbal scaled score	2.5 (1.95, range = 1–6)	3.0 (2.24) (range = 1–6)
**Current school year**		
Pre-School, *n* (%)	12 (66.7%)	3 (42.9%)
Kindergarten, *n* (%)	2 (11.1%)	2 (28.6%)
Grade 1 to 12, *n* (%)	4 (22.2%)	2 (28.6%)
**Primary caregiver highest education**		
High school and below, *n* (%)	3 (16.7%)	3 (42.9%)
College and above, *n* (%)	15 (83.4)	4 (57.1%)
**Secondary caregiver highest education**		
High school and below, *n* (%)	3 (16.7%)	1 (14.3%)
College and above, *n* (%)	13 (82.3%)	6 (85.7%)
**Social Responsiveness Scale-2 (raw scores)**		
Awareness	17.2 (2.26)	18.4 (3.15)
Cognition	29.1 (4.03)	30.3 (2.50)
Communication	49.5 (4.71)	45.7 (4.57)
Motivation	24.7 (3.82)	23.6 (4.79)
Restrictive and repetitive behaviors	31.4 (4.08)	29.4 (3.10)
Total	151.9 (12.82)	147.4 (12.41)
**Aberrant Behavior Checklist**		
Irritability	21.2 (11.61)	25.6 (14.13)
Social unresponsiveness/lethargy	6.4 (4.19)	4.0 (3.96)
Hyperactivity	15.9 (7.47)	16.0 (7.46)
Inappropriate Speech	3.0 (2.70)	3.7 (2.87)
Social Avoidance	2.4 (2.94)	2.1 (3.39)
Stereotypic Behavior	7.7 (4.50)	5.9 (3.67)
**Vineland-3 (raw scores)**		
Expressive communication	32.5 (18.29)	61.4 (24.34)
Receptive communication	43.4 (15.04)	56.9 (14.16)

*Abbreviations*: *n* = number of subjects, *SD* = standard deviation. Percentages are calculated as *n*/N*100

### Clinical measures

At visit 1, participants completed a demographic interview, BOSCC-MV, Social Responsiveness Scale-2 (SRS-2 [9];, Aberrant Behavior Checklist-Community (ABC [2];, ADOS-2 [32], and Vineland-3 [50]; see below for detailed information for each measure). At visit 2 (within 1 day of visit 1), the child was administered the BOSCC-MV and Stanford Binet 5th Edition-Abbreviated (SB-5 [45]; to determine baseline cognitive functioning. The optional visit 3 (within 6 months of visit 2) consisted of BOSCC-MV, SRS-2, ABC, ADOS-2, and Vineland-3. The SRS-2, ABC, and Vineland 3 are all caregiver report. The BOSCC-MV, Stanford Binet 5, and ADOS-2 are all administered by a clinician with the child.

### Brief observation of social communication change—minimally verbal

The BOSCC-MV coding scheme was applied to 12-min videos of free-play interactions between an adult social partner and a child. The BOSCC-MV was administered by research coordinators with bachelor’s degrees or higher and extensive experience with FXS under the supervision of one of the BOSCC-MV authors (S.H.K). The play interaction consisted of 4 min of play with a standardized set of age-appropriate toys, 2 min of bubble play, 4 min of play with a second set of toys, and another 2 min of bubble play. The BOSCC kit includes varying kinds of toys that promote multiple play levels, starting from cause and effect (e.g., Poppin’ Pals pop-up toys, musical instruments), construction (e.g., building blocks), to pretend play (e.g., pretend food items and utensils, action figures and toy vehicles). All toys also have duplicates to promote interactive play. Two different sets of BOSCC-MV kits with the same *kinds* of toys were used for visits 1 and 2; the use of the kit was counterbalanced between two visits within each site. Visits 1 day apart were considered adequate for determination of test-re-test reliability of the BOSCC-MV with alternative play boxes.

The BOSCC-MV coding scheme is comprised of 15 codes [31]. Each BOSCC-MV was scored on a 6-point scale from 0 (abnormality is not present) to 5 (abnormality is present and significantly impairs functioning). Lower scores on the BOSCC-MV denote less symptom severity. The BOSCC-MV is coded in two 6-min segments of a 12-min video (first 6-min segment A, second 6-min segment B; see [16] for more details). The administration and coding can be completed by non-clinicians (e.g., research assistants) with relatively minimal training. For the current study, two coders who achieved research reliability blind to time points of the video coded all videos under the supervision of one of the BOSCC-MV authors (S.H.K). The central coders attended an initial training workshop. After, they were deemed research reliable when they met 80% reliability on 3 consecutive videos. Their reliability was monitored throughout the study with periodical consensus coding sessions. The BOSCC-MV coding results in SC subscale and RRB subscale scores. The BOSCC-MV SC subscale score was calculated as the sum of item 1 to 8 scores. The BOSCC-MV RRB subscale score was calculated as the sum of item 9 to 13 scores. The BOSCC-MV Core score was also calculated by combining the scores of the SC and RRB subscales. The BOSCC-MV total score was calculated as the sum of all 15 item scores (see [16] for the list of items).

### Validity measures

Convergent validity of the BOSCC-MV was assessed using the Social Responsiveness Scale-2 (SRS-2 [10]; and the ADOS-2 [32]. The SRS-2 is an efficient quantitative measure of the various dimensions of interpersonal behavior, communication, and repetitive/stereotypic behavior associated with ASD. The SRS-2 school version was used for children 4 years or older, and the pre-school version was used for those under 4 years. The SRS-2 consists of 65 items that are grouped into five domains (i.e., awareness, cognition, communication, motivation, and restricted interests and repetitive behaviors), with higher scores indicating more severe impairment. Raw scores were utilized for analyses. The specific scales of interest for convergent validity were social communication and restricted interests and repetitive behaviors.

The ADOS-2 is a semi-structured, play-based diagnostic measure for ASD. Module 1, which is designed for children 31 months of age or older who are minimally verbal (no words to emerging phrases) was used in this study [32]. ADOS-2 provides algorithm scores to determine the diagnostic classification of ASD and to quantify severity of autism symptom-based behaviors during the ADOS-2 sessions. The algorithm results in two domains scores, social affect (SA) and RRB. The ADOS-2 total score was also calculated as the sum of the SA and RRB scores. Higher scores indicate higher symptom severity. Of the 18 participants, 15 met criteria for ASD on the ADOS-2.

The ABC-C [2] was also administered at study visits 1 and 3 to assess both convergent and divergent validity of the BOSCC-MV. Raw cores were analyzed using the FXS-specific factor structure such that 54 of the items resolved into 6 subscales (irritability, lethargy, social avoidance, stereotypic behavior, hyperactivity, and inappropriate speech [58];. Higher scores indicate more impairment.

The Vineland-3 (Vineland-3 [50];) is a caregiver questionnaire of their child’s adaptive behavior. The Vineland-3 consists of 49 items that are grouped into three domains (communication, daily living skills, and socialization) that correspond to the three broad domains of adaptive functioning specified by the American Association on Intellectual and Developmental Disabilities and the Diagnostic and Statistical Manual 5th edition (DSM-5). Raw scores were utilized for the Vineland-3 given the growth rate in language skills for FXS tend to be slower than the typical population, impacting standard scores as children age. Given the wide age range of this study, raw scores better provide a measure of absolute ability for the entire sample [7]. For the purposes of this analysis, only the receptive and expressive communication subscales were utilized to examine convergent validity with the BOSCC and higher scores indicate more symptom severity.

### Statistical analysis

All statistical analyses were performed using SAS, Version 9.4. Visit 1 scores were considered baseline values. Summaries of change from baseline variables included only subjects who had both visit 1 and visit 3 values. Descriptive statistics for BOSCC-MV scores was summarized by visit.

Inter-rater reliability analyses were only performed for visit 1 BOSCC-MV assessments independently coded by two raters. The inter-rater reliability of BOSCC-MV was assessed using intraclass correlation coefficients (ICCs). ICCs were calculated using a two-way random effects models with absolute agreement and single rater/measurement assumptions [39]; this methodology is equivalent to the ICC (2,1) convention described by Shrout and Fleiss [48]. Separate models were used to calculate ICCs for BOSCC-MV SC, RRB, and Core scores. ICC estimates and 95% confidence intervals were calculated for each BOSCC-MV score.

The scores from the two initial BOSCC-MV clinic administrations performed 1 day apart (visit 1 and visit 2) were used to determine test-retest reliability. ICCs were calculated using two-way random effects models with absolute agreement and single rater/measurement assumptions. Separate models were used to calculate ICCs for BOSCC-MV SC, RRB, and Core scores. ICC estimates and 95% confidence intervals were calculated for each BOSCC-MV score. For ICC interpretation, values of less than 0.5 indicate poor reliability, between 0.75 and 0.9 indicate good reliability, and values greater than 0.90 indicate excellent reliability.

Given the similarities between visit 1 and visit 2 BOSCC-MV scores, it was determined BOSCC-MV visit 1 scores would be used for all validity analyses for consistency. The scores from the first BOSCC-MV examination together with the scores from the various parent and physician reported instruments (ADOS-2, SRS-2, ABC-C, Vineland-3) were used to assess the convergent and divergent validity of BOSCC-MV. Pearson’s *r* statistic was calculated between each BOSCC-MV score at visit 1 and the various clinical outcome assessment scores. Values between 0.5 and 1 are considered strong correlations. Given the exploratory nature of this study, no corrections were made for multiple comparisons. Changes in BOSCC scores were examined for the subset of participants (*n* = 7) that returned for the third optional BOSCC-MV examination after about 6 months. Changes from baseline to optional visit 3 were summarized descriptively and tested against the null hypothesis of no change (i.e., change from baseline equal to 0) using paired *t* tests and Wilcoxon signed-rank tests for the BOSCC-MV SC, RRB, and Core scores.

## Results

### BOSCC-MV subscale scores

The mean (± SD) BOSCC-MV total score at visit 1 was 37.36 ± 9.86 (*N* = 18; Table 2) and 34.28 ± 10.05 at visit 2 (*n* = 18; Table 2). Lower scores on the BOSCC-MV indicate less symptom severity. For the SC domain, the score at visit 1 was 30.22 ± 7.81 (*N* = 18) and 28.03 ± 7.06 at visit 2 (*n* = 18; Table 2). For the RRB domain, the mean score was 5.50 ± 2.36 at visit 1 and 4.72 ± 2.87 at visit 2. For the core domain, the mean score at visit 1 was 35.72 ± 9.23 and 32.75 ± 8.78 at visit 2 (Table 2).

**Table 2:** BOSCC-MV subscale scores by visit

		Reported value Overall (*N* = 18)		
BOSCC-MV subscale	Visit	*n*	Mean (SD)	Median (min, max)
Social communication	Visit 1	18	30.22 (7.807)	33.50 (9.0, 37.5)
	Visit 2	18	28.03 (7.064)	30.25 (11.0, 37.5)
	Visit 3	7	26.36 (5.699)	25.50 (20.0, 36.5)
Restricted and repetitive behavior	Visit 1	18	5.50 (2.364)	5.25 (2.5, 9.5)
	Visit 2	18	4.72 (2.866)	4.00 (2.0, 14.0)
	Visit 3	7	5.50 (2.614)	5.00 (3.5, 11.0)
Core	Visit 1	18	35.72 (9.231)	38.00 (13.0, 47.0)
	Visit 2	18	32.75 (8.777)	33.25 (15.0, 50.0)
	Visit 3	7	31.86 (6.479)	31.50 (23.5, 42.5)
Total	Visit 1	18	37.36 (9.855)	39.25 (13.0, 49.0)
	Visit 2	18	34.28 (10.047)	33.75 (16.0, 52.5)
	Visit 3	7	32.79 (7.488)	32.00 (23.5, 45.0)

*Abbreviations*: *BOSCC-MV* = Brief Observation of Social Communication Change—Minimally Verbal, *Max* = maximum, *Min* = minimum; *n* = number of subjects, *N* = total number of subjects, *SD* = standard deviation

### Inter-rater reliability

The estimated ICC determined for the 2 expert raters at visit 1 was 0.887 (95% CI 0.705 to 0.960) for the BOSCC-MV total score across all subjects in the full analysis set. For the BOSCC-MV SC domain, inter-rater reliability was 0.873 (95%CI 0.673 to 0.955), while for the RRB domain, inter-rater reliability was slightly less at 0.713 (95% CI 0.350 to 0.892). For the BOSCC-MV Core domain, inter-rater reliability was 0.868 (95% CI 0.652 to 0.954).

### Test-retest reliability

The estimated test-retest reliability ICC measured across 2 consecutive visits (visit 1 and visit 2) was 0.764 (95% CI 0.458 or 0.906) for the BOSCC-MV total score, 0.779 (95% CI 0.488 to 0.912) for the SC domain, and 0.757 (95% CI 0.438 to 0.904) for the Core domain. For the RRB domain, test-retest reliability was lower at 0.491 (95% CI 0.065 to 0.771).

### Convergent and divergent validity

The convergent validity of the BOSCC-MV total score at visit 1 was assessed against the ADOS-2, SRS-2 Social Communication and Restricted Interests and Repetitive Behaviors, Vineland-3 Expressive and Receptive Communication subdomains, and ABC-C Social Unresponsiveness/Lethargy and Social Avoidance using Pearson’s *r* statistic (Table 3). Moderate-sized correlations were found between ADOS-2 scores and the BOSCC-MV SC (0.585–0.652) and the BOSCC-MV RRB (0.426–0.492) domains, as demonstrated in Table 3. There were weak correlations observed between the BOSCC-MV SC domain and all domains of the SRS-2 scale (Table 3). A moderate, negative correlation was identified indicating higher BOSCC-MV scores were associated with lower raw communication scores on the Vineland 3 (Table 3). All 3 BOSCC-MV domains showed small positive correlations with the ABC-C subscales social unresponsive/lethargy and social avoidance with the BOSCC-MV core domains, although none reached statistical significance (Table 3).

**Table 3:** BOSCC-MV scores at visit 1-Pearson correlation coefficients—full analysis set

		BOSCC-MV score					
		SC	*p* value	RRB	*p* value	Core	*p* value
ADOS-2	Social affect	0.615	0.009	0.426	0.088	0.623	0.008
	Restricted and repetitive behavior	0.585	0.014	0.492	0.045	0.614	0.009
	Total	0.652	0.005	0.471	0.056	0.665	0.004
SRS-2	Communication	− 0.060	0.813	0.135	0.594	− 0.016	0.949
	Restricted interests and repetitive behavior	0.109	0.668	− 0.195	0.437	0.042	0.869
ABC-C	Irritability	− 0.070	0.783	− 0.311	0.209	− 0.139	0.583
	Social unresponsiveness/lethargy	0.365	0.136	0.086	0.734	0.331	0.180
	Stereotypic behavior	0.115	0.650	− 0.288	0.247	0.024	0.926
	Hyperactivity	0.074	0.770	− 0.288	0.728	− 0.024	0.875
	Inappropriate speech	− 0.035	0.891	− 0.170	0.499	− 0.073	0.773
	Social avoidance	0.436	0.071	0.106	0.676	0.396	0.104
Vineland-3	Receptive communication	− 0.485	0.041	− 0.268	0.282	− 0.479	0.044
	Expressive communication	− 0.496	0.036	− 0.400	0.100	− 0.522	0.026

*Abbreviations*: *ABC-C* = Aberrant Behavior Checklist-Community, *ADOS-2* = Autism Diagnostic and Observation Schedule 2nd Edition, *BOSCC-MV* = Brief Observation of Social Communication Change Module 1, *RRB* = restricted and repetitive behavior; *SC* = social communication, *SRS-2* = Social Responsiveness Scale 2nd Edition, *Vineland-3* = Vineland Adaptive Behavioral Scale Comprehensive Interview Form 3rd Edition

Divergent validity was assessed against the ABC-C irritability, hyperactivity, and inappropriate speech domains and very small negative correlations were observed with all BOSCC-MV scores.

### Change in BOSCC scores over time

For the 7 subjects attending the optional visit 3, the mean BOSCC scores between visit 1 (SC = 28.36 ± 7.50, RRB = 4.21 ± 1.58, Core = 32.57 ± 8.42, total = 34.29 ± 9.09) and visit 3 (SC = 26.36 ± 5.70, RRB = 5.5 ± 2.61, Core = 31.86 ± 6.48, total = 32.79 ± 7.49) were similar for this subgroup. Using paired t-tests, there was no significant change between visits for BOSCC-MV SC (*t*(7) = 0.79, *p* = 0.46), BOSCC-MV RRB (*t*(7) = − 1.72, *p* = 0.14), BOSCC-MV Core (*t*(7) = 0.27, *p* = 0.78), or BOSCC-MV total (*t*(7) = 0.58, *p* = 0.59); similar non-significant *p* values resulted when using Wilcoxon signed-rank tests. The largest standardized difference (i.e., reported mean change divided by SD) of 0.65 was observed for the RRB subscale; standardized differences for all other subscales were less than 0.30. These results suggest relative stability in BOSCC scores over 6 months in the absence of an identified treatment or intervention.

## Discussion

Historically, treatment trials in FXS have been plagued with strong placebo effects and it has been suspected that available outcome measures are not adequately assessing change from candidate therapeutic interventions. In an effort to explore a clinician reported outcome measure of SC and RRBs for minimally verbal individuals with FXS, the BOSCC-MV was piloted with a final sample of males between the ages of 3 to 8 years with documented and genetically confirmed FXS. Males in this age group were included due to having the highest level of impairment and greatest need in the FXS population. The primary objective of this study was to examine for the first time the psychometric properties of the BOSCC-MV in minimally verbal children with FXS, specifically establishing multi-center feasibility, test-retest reliability, convergent and divergent validity, and change over an approximately 6-month period.

Overall, the BOSCC-MV was feasible to implement in a multi-site study when administered by trained research coordinators and scored by central, experienced, blinded coders. The BOSCC-MV was reliably completed across successive visits with 100% BOSCC-MV tasks being completed and codable by the central raters with all items rated for each participant. This is an important consideration for future use as a clinical outcome assessment in multicenter therapeutic clinical trials. It should be noted, that the interval between test-retest time points was quite short (i.e., a day) to lower burden on travelling families, so it should be interpreted with caution and replicated with a longer interval (e.g., a week) if possible. The sample was representative of males with FXS given the low cognitive and adaptive behavior profiles across subjects. Although we initially sought to enroll youth ages 3 to 12 for this version of the BOSCC, we were only able to enroll youth ages 3 to 8 due to advanced language in the youth screened in the 9–12 age range. Versions of the BOSCC for flexible phrase speech and complex speech are in development and may be explored in future trials for individuals who are more verbal than those in the current trial.

Test-retest for the BOSCC-MV in FXS was strong and comparable to past BOSCC-MV trials in idiopathic ASD [14, 16, 17, 28] and previous standards in idiopathic ASD measurement [11]. Reliability was similar between the BOSCC-MV SC and Core domains, while comparatively lower for the more variable BOSCC-MV RRB domain, also consistent with the results from the original psychometric papers [16, 17, 28]. These pilot results thus support that the separate domains of SC and RRB can be quantified reliably in this population. The clinical relevance of this division has been established in the idiopathic ASD literature for both the BOSCC-MV and other ASD scales [16, 18, 34, 49].

Overall inter-rater reliability was high, except on RRB which was less reliable. As mentioned above, this is very similar to the previous BOSCC-MV trials in idiopathic ASD [17] [28]. There are several possible explanations for this finding including differences in the RRB presentation in FXS, making it difficult for raters to recognize RRBs in FXS. There may also be more variability in the presence of RRBs due to phenotypic FXS characteristics including increased social anxiety and hyperactivity. The more variable test-retest values paired with the lower inter-rater reliability for RRB also suggests that RRBs may present differently in FXS versus idiopathic ASD. Reisinger et al. [43] found a peak in sensory-motor RRB severity between the ages of 2 and 12 and other RRBs between ages 7 and 12. These results may also suggest that the validity of coding of the RRBs in FXS may require specialized training in FXS and associated behaviors. Thus, future research should explore whether providing specific training to raters in commonly observed RRBs in FXS can improve the reliability of BOSCC for RRBs.

The BOSCC-MV SC domain converged with the ADOS-2 Social Affect domain, suggesting the BOSCC-MV SC domain is capturing SC difficulties in minimally verbal male children with FXS. Acceptable convergent validity was found with the ADOS-2 Social Affect domain, the Vineland-3 Receptive and Expressive Language subscales and the ABC Lethargy and Social Avoidance subscales. Yet, much smaller correlations were found between the BOSCC-MV SC scale and the SRS-2. This is consistent with past BOSCC-MV trials in idiopathic ASD, suggesting that while the SR-2 and BOSCC-MV are related, they appear to measure different aspects of SC [29]. Despite frequent use of the SRS-2 in FXS trials, there have been repeated concerns about its ability to capture SC deficits in this population. In the idiopathic ASD population, the SRS-2 has also been known to be inflated by factors other than SC such as behavioral and emotional problems [25, 26], and within FXS specifically, scores have been shown to be impacted by hyperactivity, hyperarousal, and anxiety [12]. Given the past concerns about the accuracy of SRS-2 evaluation in FXS, it is promising that the BOSCC-MV had a stronger relationship to ADOS-2 and Vineland 3 related SC scores and weaker relationship to SRS scores which were more likely to be impacted by other factors. In addition to examination of convergent validity, the relationship between measures not specifically evaluating SC was explored. No relationships were found between measures of irritability, hyperactivity, or stereotypical behavior. The lack of relationship with these measures was expected, appropriate, and provides valuable evidence of divergent validity.

In a small sample, we also examined stability and changes in the BOSCC-MV scores for the children with FXS over a 6-month period and we did not find any significant changes on the BOSCC-MV scores during this time. These results should be interpreted with caution given the sample was very small and underpowered to adequately capture sensitivity to change. It is promising that the minimal changes on the BOSCC-MV was comparable to the little change reported on other commonly used measures, such as the ABC-C, SRS-2, and Vineland-3. It is also possible that SC behaviors and RRBs are quite stable in children with FXS over a 6-month period of time in the absence of effective treatments. Sensitivity to change can only be assessed when there is change. In the small subgroup with assessments at both visit 1 and visit 3, there did not appear to be consistent changes in any endpoint over time. A larger study to allow for larger changes over time may be necessary to fully assess the sensitivity to change question. The results from this study suggest that the BOSCC-MV appears to provide reliable measures over time (whether after 1 day with different raters or over 6 months with the same rater). This is an important finding as highly variable measures are not often useful in interventional studies. In fact, stability in an outcome measure for a treatment as usual group is ideal. However, the current study was a very small sample, limiting our ability to make any definitive claims regarding the measure’s sensitivity to change. Future examinations of the BOSCC-MV as an outcome measure should have a larger sample size with children receiving both experimental treatment and treatment as usual in order to determine sensitivity to change. It may be helpful to include measures such as BOSCC-MV in longitudinal natural registries to define the natural history of FXS in younger children.

## Limitations

The sample size was very well characterized but small as this was an initial pilot trial and results must be replicated in a larger population. Particularly, the analysis of change across a longer time period will need to be further evaluated. Despite the small sample size, the results are promising for a first investigation of the BOSCC-MV in FXS which deserves further investigation as an outcome measure for SC impairment.

Given the high variability in symptom presentation within females with FXS, this pilot application only evaluated males with FXS. Despite the fact that females tend to be higher functioning overall, they often still struggle with social communication and future research should specifically evaluate the utility of the BOSCC-MV for severely impaired females. It is likely that future versions of the BOSCC for individuals with more advanced language will need to be utilized for the majority of females, given they tend to have stronger language and communication abilities.

## Conclusions

Recent BOSCC-MV idiopathic ASD trials have utilized different contexts to apply the measure such as play and snack interactions with caregivers, suggesting that the BOSCC-MV captures similar change in SC across situations [14]. Given the possible confounding impacts of anxiety and hyperarousal in FXS, future trials should explore potential differences in SC with a known caregiver in a more naturalistic environment versus a lab setting with a research coordinator. It is suspected that these different contexts will provide both valuable information about the impact of context as well as a more accurate assessment of social communication in the FXS population.

Overall, the promising reproducibility, convergence with other known social communication assessment measures, feasibility for multicenter settings using a central reader, and divergence with measures not assessing social communication support further evaluation in trials with larger sample sizes to assess the utility of the BOSCC-MV in FXS with severe communication impairment. Further research into the use of the BOSCC-MV in this population may be explored with larger, independent samples. The other versions of the BOSCC for verbal individuals should also be evaluated with samples with more advanced verbal ability.

## Data Availability

Data is not available for this project due to confidentiality concerns with the BOSCC-MV videos. The BOSCC-MV materials are under copyright with WPS.

## Abbreviations

FXS: Fragile X syndrome
ASD: Autism spectrum disorder
ADOS-2: Autism Diagnostic and Observation Schedule
BOSCC-MV: Brief Observation of Social Communication Change—Minimally Verbal
SC: Social Communication
RRB: Restricted and Repetitive Behaviors
ABC-2: Aberrant Behavior Checklist, 2
SRS: Social Responsiveness Scale

## References

[1] Abbeduto L, Brady N, Kover ST (2007). Language development and fragile X syndrome: profiles, syndrome-specificity, and within-syndrome differences. Ment Retard Dev Disabil Res Rev.

[2] Aman MG, Singh NN, Stewart AW, Field CJ (1985). The Aberrant Behavior Checklist: A behavior rating scale for the assessment of treatment effects. Am J Mental Deficiency.

[3] Benjamin DP, McDuffie AS, Thurman AJ, Kover ST, Mastergeorge AM, Hagerman RJ, Abbeduto L (2015). Effect of speaker gaze on word learning in fragile X syndrome: a comparison with nonsyndromic autism spectrum disorder. J Speech Lang Hear Res.

[4] Berry-Kravis E, Des Portes V, Hagerman R, Jacquemont S, Charles P, Visootsak J, von Raison F (2016). Mavoglurant in fragile X syndrome: results of two randomized, double-blind, placebo-controlled trials. Sci Transl Med.

[5] Boyle L, Kaufmann WE (2010). The behavioral phenotype of FMR1 mutations. Am J Med Genet C Semin Med Genet.

[6] Budimirovic DB, Berry-Kravis E, Erickson CA, Hall SS, Hessl D, Reiss AL, Kaufmann WE (2017). Updated report on tools to measure outcomes of clinical trials in fragile X syndrome. J Neurodev Disord.

[7] Channell MM, Thurman AJ, Kover ST, Abbeduto L (2014). Patterns of change in nonverbal cognition in adolescents with Down syndrome. Res Dev Disabil.

[8] Coghlan S, Horder J, Inkster B, Mendez MA, Murphy DG, Nutt DJ (2012). GABA system dysfunction in autism and related disorders: from synapse to symptoms. Neurosci Biobehav Rev.

[9] Constantino JN (2012). Social Responsiveness Scale.

[10] Constantino JN, Gruber CP (2005). Social responsiveness scale.

[11] Cunningham AB (2012). Measuring change in social interaction skills of young children with autism. J Autism Dev Disord.

[12] Factor RS, Ryan SM, Farley JP, Ollendick TH, Scarpa A (2017). Does the presence of anxiety and ADHD symptoms add to social impairment in children with autism spectrum disorder?. J Autism Dev Disord.

[13] Flenthrope JL, Brady NC (2010). Relationships between early gestures and later language in children with fragile X syndrome. Am J Speech Lang Pathol.

[14] Frost KM, Koehn GN, Russell KM, Ingersoll B (2019). Measuring child social communication across contexts: Similarities and differences across play and snack routines. Autism Res.

[15] Gallagher A, Hallahan B (2012). Fragile X-associated disorders: a clinical overview. J Neurol.

[16] Grzadzinski R, Carr T, Colombi C, McGuire K, Dufek S, Pickles A, Lord C (2016). Measuring changes in social communication behaviors: preliminary development of the Brief Observation of Social Communication Change (BOSCC). J Autism Dev Disord.

[17] Grzadzinski R, Janvier D, Kim SH (2020). Recent developments in treatment outcome measures for young children with autism spectrum disorder (ASD). Seminars Pediatric Neurol.

[18] Guthrie W, Swineford LB, Wetherby AM, Lord C (2013). Comparison of DSM-IV and DSM-5 factor structure models for toddlers with autism spectrum disorder. J Am Acad Child Adolesc Psychiatry.

[19] Hagerman RJ, Berry-Kravis E, Kaufmann WE, Ono MY, Tartaglia N, Lachiewicz A, Visootsak J (2009). Advances in the treatment of fragile X syndrome. Pediatrics.

[20] Hahn LJ, Brady NC, McCary L, Rague L, Roberts JE (2017). Early social communication in infants with fragile X syndrome and infant siblings of children with autism spectrum disorder. Res Dev Disabil.

[21] Hall SS, Lightbody AA, McCarthy BE, Parker KJ, Reiss AL (2012). Effects of intranasal oxytocin on social anxiety in males with fragile X syndrome. Psychoneuroendocrinology.

[22] Hall SS, Lightbody AA, Reiss AL (2008). Compulsive, self-injurious, and autistic behavior in children and adolescents with fragile X syndrome. Am J Ment Retard.

[23] Hall SS, Monlux KD, Rodriguez AB, Jo B, Pollard JS (2020). Telehealth-enabled behavioral treatment for problem behaviors in boys with fragile X syndrome: a randomized controlled trial. J Neurodev Disord.

[24] Harkins, C. M., Dominick, K. C., Wink, L. K., Pedapati, E. V., Shaffer, R. C., Fitzpatrick, S. E., . . . Erickson, C. A. Challenges in conducting clinical trials for pharmacotherapies in Fragile X syndrome: Lessons Learned. Pharmaceutical Medicine. 2017 10.1007/s40290-017-0199-1

[25] Havdahl KA, Hus Bal V, Huerta M, Pickles A, Øyen AS, Stoltenberg C, Bishop SL (2016). Multidimensional influences on autism symptom measures: implications for use in etiological research. J Am Acad Child Adolesc Psychiatry.

[26] Hus V, Bishop S, Gotham K, Huerta M, Lord C (2013). Factors influencing scores on the social responsiveness scale. J Child Psychol Psychiatry.

[27] Jones RM, Carberry C, Hamo A, Lord C (2017). Placebo-like response in absence of treatment in children with Autism. Autism Res.

[28] Kim SH, Grzadzinski R, Martinez K, Lord C (2019). Measuring treatment response in children with autism spectrum disorder: applications of the Brief Observation of Social Communication Change to the Autism Diagnostic Observation Schedule. Autism.

[29] Kitzerow J, Teufel K, Wilker C, Freitag CM (2016). Using the brief observation of social communication change (BOSCC) to measure autism-specific development. Autism Res.

[30] Kover ST, Abbeduto L (2010). Expressive language in male adolescents with fragile X syndrome with and without comorbid autism. J Intellect Disabil Res.

[31] Lord C, Grzadzinski R, Holbrook A, Kim SH (2020). Brief Observation of Social Communication Change (BOSCC).

[32] Lord C, Luyster RJ, Gotham K, Gutherie W (2012). Autism diagnostic and observation schedule, second edition (ADOS-2).

[33] Losh M, Martin GE, Klusek J, Hogan-Brown AL (2012). Pragmatic Language in autism and fragile X syndrome: genetic and clinical applications. Perspect Lang Learn Educ.

[34] Mandy WP, Charman T, Skuse DH (2012). Testing the construct validity of proposed criteria for DSM-5 autism spectrum disorder. J Am Acad Child Adolesc Psychiatry.

[35] Marschik PB, Bartl-Pokorny KD, Sigafoos J, Urlesberger L, Pokorny F, Didden R, Kaufmann WE (2014). Development of socio-communicative skills in 9- to 12-month-old individuals with fragile X syndrome. Res Dev Disabil.

[36] Martin GE, Roberts JE, Helm-Estabrooks N, Sideris J, Vanderbilt J, Moskowitz L (2012). Perseveration in the connected speech of boys with fragile X syndrome with and without autism spectrum disorder. Am J Intellect Dev Disabil.

[37] McDuffie A, Banasik A, Bullard L, Nelson S, Feigles RT, Hagerman R, Abbeduto L (2018). Distance delivery of a spoken language intervention for school-aged and adolescent boys with fragile X syndrome. Dev Neurorehabil.

[38] McDuffie A, Thurman AJ, Hagerman RJ, Abbeduto L (2015). Symptoms of Autism in Males with Fragile X Syndrome: A Comparison to Nonsyndromic ASD Using Current ADI-R Scores. J Autism Dev Disord.

[39] McGraw KO, Wong SP (1996). Forming inferences about some intraclass correlation coefficients. Psychological Methods.

[40] Moss J, Oliver C, Arron K, Burbidge C, Berg K (2009). The prevalence and phenomenology of repetitive behavior in genetic syndromes. J Autism Dev Disord.

[41] Murphy MM, Abbeduto L (2007). Gender differences in repetitive language in fragile X syndrome. J Intellect Disabil Res.

[42] Peprah E (2012). Fragile X syndrome: the FMR1 CGG repeat distribution among world populations. Ann Hum Genet.

[43] Reisinger DL, Shaffer RC, Tartaglia N, Berry-Kravis E, Erickson CA. Delineating repetitive behavior profiles across the lifespan in fragile X syndrome. Brain Sci. 2020;10(4). 10.3390/brainsci10040239. 10.3390/brainsci10040239 PMC7226450 32316611

[44] Roberts J, Martin GE, Moskowitz L, Harris AA, Foreman J, Nelson L (2007). Discourse skills of boys with fragile X syndrome in comparison to boys with Down syndrome. J Speech Lang Hear Res.

[45] Roid G (2003). Stanford-Binet Intelligence Scales.

[46] Schneider A, Hagerman RJ, Hessl D (2009). Fragile X syndrome -- from genes to cognition. Dev Disabil Res Rev.

[47] Shaffer, R. C., Schmitt, L., John Thurman, A., Abbeduto, L., Hong, M., Pedapati, E., . . . Erickson, C. The relationship between expressive language sampling and clinical measures in fragile X syndrome and typical development. Brain Sci, 2020; 10(2). 10.3390/brainsci10020066 10.3390/brainsci10020066 PMC7071383 31991905

[48] Shrout PE, Fleiss JL (1979). Intraclass correlations: uses in assessing rater reliability. Psychological Bulletin.

[49] Shuster J, Perry A, Bebko J, Toplak ME (2014). Review of factor analytic studies examining symptoms of autism spectrum disorders. J Autism Dev Disord.

[50] Sparrow S, Cicchetti D, Saulnier C (2016). Vineland Adaptive Behavior Scale-Third Edition (Vineland-3).

[51] Sudhalter V, Belser RC. Conversational characteristics of children with fragile X syndrome: tangential language. Am J Ment Retard. 2001;106(5):389–400. doi:10.1352/0895- 8017(2001)106<0389:Ccocwf>2.0.Co;2. 10.1352/0895-8017(2001)106<0389:CCOCWF>2.0.CO;2 11531459

[52] Thurman AJ, McDuffie A, Hagerman R, Abbeduto L (2014). Psychiatric symptoms in boys with fragile X syndrome: a comparison with nonsyndromic autism spectrum disorder. Res Dev Disabil.

[53] Thurman AJ, McDuffie A, Hagerman RJ, Josol CK, Abbeduto L (2017). Language skills of males with fragile X syndrome or nonsyndromic autism spectrum disorder. J Autism Dev Disord.

[54] Thurman AJ, McDuffie A, Kover ST, Hagerman R, Channell MM, Mastergeorge A, Abbeduto L (2015). Use of emotional cues for lexical learning: a comparison of autism spectrum disorder and fragile X syndrome. J Autism Dev Disord.

[55] Thurman AJ, McDuffie A, Kover ST, Hagerman RJ, Abbeduto L (2015). Autism symptomatology in boys with fragile X syndrome: a cross sectional developmental trajectories comparison with nonsyndromic autism spectrum disorder. J Autism Dev Disord.

[56] Thurman AJ, Potter LA, Kim K, Tassone F, Banasik A, Potter SN, Abbeduto L (2020). Controlled trial of lovastatin combined with an open-label treatment of a parent-implemented language intervention in youth with fragile X syndrome. J Neurodev Disord.

[57] Weber JD, Smith E, Berry-Kravis E, Cadavid D, Hessl D, Erickson C. Voice of people with fragile X syndrome and their families: reports from a survey on treatment priorities. Brain Sci. 2019;9(2). 10.3390/brainsci9020018. 10.3390/brainsci9020018 PMC6406416 30678024

[58] Wheeler A, Raspa M, Bann C, Bishop E, Hessl D, Sacco P, Bailey DB (2014). Anxiety, attention problems, hyperactivity, and the Aberrant Behavior Checklist in fragile X syndrome. Am J Med Genet A.

[59] Wolff JJ, Bodfish JW, Hazlett HC, Lightbody AA, Reiss AL, Piven J (2012). Evidence of a distinct behavioral phenotype in young boys with fragile X syndrome and autism. J Am Acad Child Adolesc Psychiatry.

[60] Youssef EA, Berry-Kravis E, Czech C, Hagerman RJ, Hessl D, Wong CY, Quiroz JA (2018). Effect of the mGluR5-NAM basimglurant on behavior in adolescents and adults with fragile X syndrome in a randomized, double-blind, placebo-controlled trial: FragXis phase 2 results. Neuropsychopharmacol.

